# The Role of Organokines in Obesity and Type 2 Diabetes and Their Functions as Molecular Transducers of Nutrition and Exercise

**DOI:** 10.3390/metabo13090979

**Published:** 2023-08-29

**Authors:** Ji Ye Lim, Eunju Kim

**Affiliations:** Department of Biochemistry and Molecular Biology, McGovern Medical School, The University of Texas Health Science Center at Houston (UTHealth), 6431 Fannin St., Houston, TX 77030, USA

**Keywords:** hepatokines, myokines, adipokines, metabolic disease, type 2 diabetes, obesity, nutrition, exercise

## Abstract

Maintaining systemic homeostasis requires the coordination of different organs and tissues in the body. Our bodies rely on complex inter-organ communications to adapt to perturbations or changes in metabolic homeostasis. Consequently, the liver, muscle, and adipose tissues produce and secrete specific organokines such as hepatokines, myokines, and adipokines in response to nutritional and environmental stimuli. Emerging evidence suggests that dysregulation of the interplay of organokines between organs is associated with the pathophysiology of obesity and type 2 diabetes (T2D). Strategies aimed at remodeling organokines may be effective therapeutic interventions. Diet modification and exercise have been established as the first-line therapeutic intervention to prevent or treat metabolic diseases. This review summarizes the current knowledge on organokines secreted by the liver, muscle, and adipose tissues in obesity and T2D. Additionally, we highlighted the effects of diet/nutrition and exercise on the remodeling of organokines in obesity and T2D. Specifically, we investigated the ameliorative effects of caloric restriction, selective nutrients including ω3 PUFAs, selenium, vitamins, and metabolites of vitamins, and acute/chronic exercise on the dysregulation of organokines in obesity and T2D. Finally, this study dissected the underlying molecular mechanisms by which nutrition and exercise regulate the expression and secretion of organokines in specific tissues.

## 1. Introduction

Over the past few decades, the rates of obesity and type 2 diabetes (T2D) have increased worldwide [1]. It is well-known that obesity is an established contributor to T2D, as well as a core component of metabolic syndrome [2,3]. Sedentary lifestyle and the consumption of high-calorie diets are associated with increased risks of obesity and metabolic syndrome, ultimately leading to T2D [1,4,5,6]. At the same time, the level of sedentariness and diet quality are affected by various factors, including stress [7,8]. In this regard, numerous studies have shown that elevated psychological distress is associated with physical inactivity and a high consumption of unhealthy foods, such as foods with a high glycemic index, ultra-processed foods, and snack-type foods, as well as a low consumption of meat, fish, fruits, and vegetables [8,9]. These lifestyle risk factors heavily increase the risks of obesity, metabolic syndrome, and T2D [1,4,5,6]. Therefore, lifestyle modifications have emerged as an appealing approach to treating obesity and T2D.

To maintain energy homeostasis, a complex and delicate network between organs has evolved in higher organisms. Carbohydrates and lipids are the two critical macromolecules that are key components of intracellular storage products for energy production [10]. The metabolism of these macromolecules is interwoven in insulin-sensitive organs, including the liver, muscle, and adipose tissues [10]. Insulin resistance, fat accumulation, and inflammation in these tissues characterize metabolic diseases, such as T2D and obesity. Recently, obesity and T2D have been considered multifactorial and complex metabolic diseases resulting from alterations in the metabolic interorgan crosstalk [11,12]. Interorgan crosstalk can be defined as the broad effects of secreted factors from tissues that may trigger physiological responses in other tissues, affecting homeostasis or the development of diseases [11,13]. Interorgan crosstalk is known to be governed by hormones and metabolites. Nevertheless, recent evidence suggests that organokines are crucial factors in interorgan crosstalk [14,15,16]. Organokines (including myokines, adipokines, and hepatokines) are proteins prominently secreted from specific organs and have been known to have endocrine or paracrine actions [17].

The liver is the major insulin-sensitive organ, which is crucial in regulating energy metabolism, including lipid and glucose metabolisms. In T2D and obesity conditions where insulin resistance is manifested, impaired insulin activity increases endogenous hepatic glucose production and decreases glucose uptake, thereby leading to glycotoxicity in organs such as adipose tissues and muscle [18,19]. Recent evidence indicated that hepatokines affect fat and muscle metabolic phenotypes in an endocrine-dependent manner, and muscle and fat metabolic phenotypes in an endocrine-dependent manner [20]. Furthermore, hyperglycemia in patients with T2D markedly increased de novo hepatic lipogenesis, leading to hepatic insulin resistance, hepatic inflammation, non-alcoholic fatty liver disease (NAFLD), and severe liver diseases such as cirrhosis and hepatocellular carcinoma [21]. Among metabolic disorders, T2D and NAFLD are multi-system diseases that impair the ability of peripheral tissues to communicate to regulate lipid homeostasis and excessive cytokine release from the liver, adipose tissues, and the skeletal muscle [22,23].

Skeletal muscle is one of the largest organs in humans without obesity, comprising approximately 30–40% of the body weight in lean men and women. Skeletal muscle cells have been regarded as secretory cells [24] as they can produce and secrete myokines into circulation, exerting endocrine effects on other organs such as the liver and adipose tissues. As most myokine production is intimately related to muscle contraction, physical inactivity/activity can remodel the myokine profile and its responses [24]. Cytokines, including myokines, which are produced in response to acute or chronic exercise, are often referred to as exerkines [25]. Exerkines are secreted in many forms, including hormones, metabolites, proteins, and nucleic acids, by muscle and other metabolically active tissues [25]. Mounting evidence suggests that exerkines are beneficial in improving systemic metabolic health such as T2D, obesity, and cardiovascular disease [25].

Adipose tissue was considered a storage organ but has now been recognized as an endocrine organ [26]. Excess adiposity and adipose tissue dysfunction are associated with various metabolic diseases such as T2D, obesity, and atherosclerosis [27]. White adipose tissue (WAT) is an insulin-sensitive organ that secretes adipokines that modulate energy balance levels in other organs [28]. The main WAT includes subcutaneous and visceral adipose tissue, which have specific expression patterns of adipokines. For example, visceral WAT is related to the pathophysiology of obesity, dyslipidemia, and insulin resistance, while subcutaneous adipose tissue is related to insulin sensitivity [29,30,31].

Exercise, which is regarded as physical activity, includes resistance training, aerobic training, or high-intensity training [32]. It is a well-established notion that exercise is a critical intervention for decreasing the incidence and prevalence of multiple diseases [25]. Exercise promotes myokine secretion from the muscle tissues and broadly affects the liver, WAT, and heart [25]. These organokines, which are produced in response to exercise, are increasingly recognized as targets for treating metabolic diseases. Moreover, the production of organokines, including hepatokines, myokines, and adipokines, is also prominently affected by the diet (macro- and micronutrients). Therefore, understanding the molecular mechanisms by which exercise and diet affect organokines in the body is crucial in improving disease pathophysiology.

This review introduces the importance of organokines, including hepatokines, myokines, and adipokines, in the pathophysiology of metabolic diseases, specifically focusing on obesity and T2D. Furthermore, the current review also suggests that exercise and nutrients/diets can be beneficial strategies in remodeling organokines and alleviating the pathophysiology of obesity and T2D.

## 2. Hepatokines

### 2.1. α2-HS-Glycoprotein (Fetuin-A)

Fetuin-A is a circulating glycoprotein that is abundantly synthesized and secreted from the adipose tissue and the liver [33]. It is a multifaceted molecule functioning in various molecular pathways, including insulin resistance, inflammation, and calcium and bone metabolism [34]. Accumulating epidemiologic evidence suggests that elevated Fetuin-A is associated with obesity [35] and T2D [36]. Reportedly, hyperenergetic, high-fat diet consumption leads to Fetuin-A mRNA synthesis in rats [37] and elevated Fetuin-A in healthy men [38]. Furthermore, palmitate treatment stimulates nuclear factor- κB binding to the promoter region of Fetuin-A [39]. Fetuin-A is a critical inhibitor of insulin receptor tyrosine kinase autophosphorylation, which impairs insulin signaling [40,41]. Many studies observed a positive association between serum Fetuin-A and insulin resistance, risk of T2D, and impaired glucose tolerance [36,42,43]. In mice, the deletion of fetuin led to enhanced insulin sensitivity and glucose clearance with insulin-stimulated phosphorylation of insulin receptor kinase and downstream signaling pathways such as MAPK and Akt in skeletal muscle and liver tissues [44]. Apart from its impact on insulin receptor kinase, Fetuin-A is known to propagate a pro-inflammatory state, promoting insulin resistance. In both monocytes and adipocytes, Fetuin-A treatment significantly increased the expression of pro-inflammatory cytokines with a significant reduction in adiponectin expression [39,45]. Moreover, Fetuin-A is an endogenous ligand that directly binds to toll-like receptor (TLR) 4 and promotes the TLR4-mediated inflammatory signaling pathway [46]. These results suggest that targeting Fetuin-A is a potential therapeutic strategy for treating insulin resistance and T2D.

### 2.2. Fibroblast Growth Factor 21 (FGF21)

FGF21, predominantly expressed and synthesized in the liver, is suggested as a stress-responsive metabolic regulator [47]. Triglyceride (TG) has been suggested to be a key driver of FGF21 expression in the liver [48]. In humans, plasma levels of FGF21 are known to be increased with insulin resistance in the liver and muscles [49]. Similarly, patients with T2D exhibited an increased level of FGF21 [50], which may be due to the compensatory response for insulin deficiency. Endogenous FGF21 is induced in obese mice and humans in response to elevated cellular stress. Despite high levels of FGF21 in metabolic disease states in the body, exogenous FGF21 administration has been demonstrated to be beneficial in correcting dysregulated metabolism [51]. Intensive research uncovered that systemic administration of high doses of FGF21 strongly prevents or treats metabolic diseases [52,53,54,55]. Similar studies utilizing transgenic mice overexpressing FGF21 mice or FGF21 analog-treated mice showed improvements in multiple metabolic parameters, such as body weight gain, blood glucose, circulating inflammatory cytokines, and adipokines [52,56].

Furthermore, long-term production of FGF21 via administration of AAV8-pAAT-FGF21 targeting the liver reversed the hallmarks of obesity and T2D in high-fat diet-fed mice [50]. A recent clinical trial showed that FGF21 analog treatment for 12 weeks significantly improved lipid profiles with increased adiponectin levels in patients with obesity and T2D [57]. FGF21 improves obesity and insulin resistance by regulating several molecular mechanisms. FGF21 activates the AMPK signaling pathway, suppressing de novo lipogenesis and enhancing fatty acid oxidation, thereby inhibiting TG accumulation in adipose tissues [58]. FGF21 increases browning of WAT by up-regulating uncoupling protein 1 (UCP1) and promotes TG turnover [59]. Notably, FGF21 accelerates TG-derived free fatty acid uptake by tissues by up-regulating low-density lipoprotein receptor (LDLR) expression [60]. There are several known downstream targets of FGF21, such as AMPK and SIRT1 [58]. However, future studies are required to elucidate the downstream targets of FGF21, which possesses the potential of therapeutic targets to treat obesity and T2D.

### 2.3. Leukocyte Cell-Derived Chemotaxin 2 (LECT2)

Leukocyte cell-derived chemotaxin 2 (LECT2) is a recently discovered hepatokine that plays crucial roles in various diseases, such as obesity [61], T2D [62], liver fibrosis [63], and hepatocellular carcinoma [64]. Studies have reported that hepatic and serum levels of LECT2 are known to be strongly associated with BMI and liver fat in humans [62] and mice [65]. A recent mechanistic study showed that LECT2 treatment markedly suppressed insulin signaling by decreasing the levels of insulin receptor substrate (IRS) and p-Akt in differentiated 3T3-L1 cells [66]. Moreover, LECT2 treatment led to a significant increase in lipid accumulation and inflammation markers, such as NF-κB in 3T3-L1 cells [66]. LECT2 also targets skeletal muscle and causes insulin resistance by activating the c-Jun N-terminal kinase (JNK) pathway in obesity [62]. Conversely, LECT2 knockout mice are strongly protected from developing obesity and liver inflammation following high-fat diet feeding [61]. Additionally, LECT2 knockout mice exhibited a significantly higher level of muscle endurance compared with that in control mice [62]. These studies suggest that LECT2 can be a molecular target to enhance insulin signaling in multiple tissues, such as WAT and muscles.

### 2.4. Selenoprotein P

Selenoprotein P (SeP) is a widely present extracellular glycoprotein [67,68], and its levels are found to be increased in individuals with conditions, such as NAFLD, T2D, and visceral obesity [69,70]. The liver is the primary site of SeP production, and it is subsequently released into the bloodstream. Studies involving patients with T2D have shown that hepatic SeP expression is associated with insulin resistance [71]. Moreover, when SeP was administered to mice, it hindered insulin signaling and inhibited AMPK activation in the liver. Conversely, depleting SeP in mice resulted in improved insulin sensitivity and glucose tolerance [72].

## 3. Myokines

### 3.1. IL-6

Interleukin-6 (IL-6), which is secreted into the bloodstream during muscle contractions, was the first myokine discovered [73]. It is primarily produced by skeletal muscle and its release into the blood increases significantly during exercise, reaching levels up to 100-fold higher than that at rest [74]. Interestingly, IL-6 exerts varying and diverse effects in the body. It promotes myogenic differentiation within skeletal muscle itself and increases glucose uptake by facilitating the translocation of glucose transporter type 4 (GLUT4), a glucose transporter [75,76]. IL-6 also activates 5′ AMP-activated protein kinase (AMPK), an enzyme involved in energy metabolism, leading to increased fatty acid oxidation in both skeletal muscle and adipose tissue [77,78,79]. Of note, IL-6 secretion influences hepatic glucose production, specifically in the regulation of gluconeogenesis, by inducing the expression of PCK1 [80]. In addition, IL-6 secretion is inversely related to plasma glucose levels, suggesting its role in inducing glycogenolysis in the liver under conditions such as overnight fasting when circulating glucose is limited [80]. The effect of IL-6 on glucose production depends on the metabolic state. Notably, mice lacking IL-6 exhibit obesity and glucose intolerance, indicating the beneficial role of muscle-derived IL-6 in metabolic regulation [81].

Another important aspect is the muscle–liver crosstalk, where the exercise-induced expression of CXCL1 is dependent on IL-6 secretion from skeletal muscles [80]. IL-6 plays a crucial role in liver homeostasis. However, its secretion by activated Küpffer cells in the liver is mostly associated with the acute phase of infection [82]. Some studies have shown that IL-6 can impair insulin signaling and disrupt glucose homeostasis through signaling pathways such as JNK1, STAT3, and SOCS3 [83,84,85]. In addition to its metabolic effects, IL-6 also functions as an anti-inflammatory factor by inhibiting the production of tumor necrosis factor (TNF) [81,86,87]. Conversely, in the absence of IL-6, the levels of TNF are elevated. These findings suggest that muscle-derived IL-6 plays a beneficial role in the regulation of metabolic disorders, affecting glucose production during exercise [88]. Moreover, IL-6 induces the production of another cytokine, CXCL-1, which is involved in hepatic regulation during exercise [89]. These findings have suggested a bimodal and pleiotropic role for IL-6 in liver homeostasis, indicating the need for further research using different approaches, such as cell-specific depletion or varying doses of recombinant proteins, to better understand the precise role of IL-6 signaling in the liver, particularly as a myokine.

### 3.2. Irisin

Irisin is a myokine that acts as a transcriptional co-activator of PGC1α, a master regulator of muscle energy metabolism that is involved in mitochondrial biogenesis, glucose uptake, and fiber-type switching [90,91,92]. The levels of circulating irisin levels have been positively associated with muscle mass, and negatively associated with fat mass, indicating the role of irisin in exercise-induced metabolic adaptation [90]. Following the cleavage of FNDC5, irisin is released from the muscle and circulates in the bloodstream, where it targets white adipocytes in WAT, inducing their transformation into beige cells, and hence leading to increased energy expenditure and potential weight loss [90,93]. The adipose tissue has been recently identified as a source of irisin secretion, with individuals with obesity tending to have higher levels of circulating irisin [94,95,96]. Furthermore, the levels of circulating irisin in humans have been positively correlated with adiposity parameters and insulin resistance markers [96]. Irisin reduces insulin resistance by inhibiting gluconeogenesis and promoting glycogenesis via the PI3K/AKT/FOXO1 pathway in hepatocytes [97]. In conclusion, irisin has multifaceted effects on liver metabolism, including reducing insulin resistance, lipogenesis, and oxidative stress, while promoting glycogenesis and fatty acid oxidation. However, its impact on humans and its role in various metabolic conditions require further investigation to clarify its therapeutic potential and clinical application.

### 3.3. FGF21

FGF21 is a myokine produced by skeletal muscles that exerts various metabolic functions, particularly in the liver [98,99,100]. It enhances glucose uptake and increases the expression of GLUT1 in skeletal muscle [101]. Activation of the phosphoinositide 3-kinase/protein kinase B (PI3K/AKT1) signaling pathway, which is linked to muscle hypertrophy, leads to increased muscle mass, reduced fat mass, and improved overall energy metabolism. Insulin infusion and exercise have been shown to increase the expression and secretion of muscular FGF21 [98,99]. Additionally, the induced expression of FGF21 in muscles has been associated with increased lipolysis, decreased blood glucose levels, enhanced fatty acid oxidation, and WAT browning [102]. Another piece of evidence reinforcing the idea that FGF21 is a myokine was shown to be the increased expression of FGF21 in mice with skeletal muscle-specific overexpression of UCP1 [103]. These findings supported the role of FGF21 as a myokine, which has potential therapeutic implications in T2D and obesity.

### 3.4. IL-15

IL-15, which is a member of the IL-2 superfamily, is putative myokine produced by skeletal muscles, exhibiting anabolic effects in this tissue [104]. Its levels increase in both muscles and serum after strength training [105,106,107,108]. IL-15 is involved in skeletal muscle growth and has been closely associated with obesity and T2D [109,110]. It enhances glucose uptake in skeletal muscle by increasing the transcription and membrane translocation of GLUT4 through JAK3/STAT3 signaling [111,112]. IL-15 also enhances the activity of PPARδ and PGC-1α, promoting mitochondrial biogenesis and fatty acid oxidation (FAO) in skeletal muscle [113,114,115]. In addition, it has been shown to decrease lipid deposition in preadipocytes and overall WAT mass [104]. Although its presence in the plasma has not been observed, IL-15 might act as a myokine, regulating WAT homeostasis [116,117]. This hypothesis is supported by studies showing an inverse correlation between IL-15, adipose tissue mass, and abdominal adiposity in humans [117]. Overexpression of IL-15 in mouse muscles was reported to reduce visceral fat mass without affecting subcutaneous fat mass [117]. Elevated plasma levels of IL-15 significantly decreased body fat mass without affecting lean body mass or other cytokine levels in mice [117]. These findings indicated that muscle-secreted IL-15 reduces visceral fat mass through the endocrine system, highlighting the role of the muscle–fat crosstalk.

### 3.5. FSTL

Follistatin (FSTL) is a member of the TGF-β superfamily that serves as a natural inhibitor of myostatin in skeletal muscles [118]. In a mouse model, swimming exercise significantly increased the levels of follistatin in both the plasma and liver tissue. Elevated levels of circulating follistatin were reported to play a role in regulating myostatin levels in skeletal muscles [119]. FSTL-1 is one of the secreted glycoproteins belonging to the follistatin family [120]. Myogenic AKT, a key factor in blood vessel growth and muscle growth, plays a significant role in regulating FSTL-1 [121]. Overexpression of AKT, specifically in the muscle, was shown to lead to increased intramuscular and circulating serum levels of FSTL-1 in both intramuscular and circulating serum. Elevated FSTL-1 levels enhanced endothelial function and revascularization by activating the AKT-eNOS signaling pathway. In human primary skeletal muscle cells, the expression and secretion of FSTL-1 were found to be increased in a differentiation-dependent manner [120]. Furthermore, exercise has been shown to increase the circulating levels of FSTL-1, with the secretion of FSTL-1 being stimulated by IFNγ and IL-1β [120].

## 4. Adipokines

### 4.1. Leptin

Leptin is a well-known adipokine primarily secreted by adipocytes into the bloodstream. The levels of leptin in the body have been directly associated with fat mass [122]. In individuals with obesity, the levels of leptin positively correlate with adipose tissue mass, suggesting leptin as a marker of obesity [123]. For instance, Ob/ob mice exhibit characteristics such as increased food intake, decreased energy expenditure, dyslipidemia, obesity, and insulin resistance [124,125]. Leptin also promotes inflammation by enhancing the production of inflammatory cytokines, such as TNF and IL-6 by monocytes, stimulating the generation of reactive oxygen species (ROS), and inducing cell proliferation and migration in monocytes [123,126,127]. In macrophages, leptin activates the JAK2/STAT3 signaling pathway, leading to the production of CC-chemokine ligands [128]. Chronic inflammation and elevated TNFα levels in individuals with obesity and leptin resistance contribute to hyperleptinemia [129,130].

### 4.2. Adiponectin

Adiponectin is a hormone secreted by mature adipocytes that is inversely correlated with fat mass [131]. Unlike leptin, higher levels of adiponectin have been associated with lower fat mass [131]. The plasma levels of adiponectin are found to be reduced in individuals with obesity, type II diabetes, and insulin resistance, exhibiting an inverse correlation between the levels of adiponectin and BMI [132,133,134]. Adiponectin signaling is involved in increasing insulin sensitivity and has various beneficial effects on metabolism, including reducing adiposity, inflammation, and atherosclerosis [135,136,137]. It affects different target organs, such as the liver, where it decreases gluconeogenesis and insulin resistance [138,139]; skeletal muscle, where it enhances fatty acid oxidation, glucose uptake, and mitochondrial biogenesis [140,141]; and the brain, where it stimulates energy expenditure [136]. Adiponectin also plays a role in enhancing glucose uptake and fatty acid oxidation in skeletal muscle and suppressing glucose production in the liver through the activation of AMPK [141,142]. Moreover, adiponectin stimulates insulin secretion, whereas its deficiency leads to dysfunction of pancreatic β-cells [143]. The expression of adiponectin by adipocytes is decreased in individuals with obesity, while it is also inhibited by pro-inflammatory cytokines, such as TNF and IL-6, as well as conditions such as hypoxia and oxidative stress [144,145,146].

### 4.3. TNFα

Tumor necrosis factor-α (TNFα) is an inflammatory cytokine predominantly produced by monocytes and macrophages. In individuals with obesity, the macrophage-infiltrated visceral fat becomes a major source of TNFα production [147,148]. Increased expression of TNFα has been observed in the adipose tissue of humans and mouse models of obesity and T2D [149,150]. In *ob/ob* mice, deletion of TNFα or obesogenic diet leads to a reduction in insulin resistance and improvement in insulin signaling in the WAT and muscles [150]. Furthermore, this cytokine has been associated with reduced insulin sensitivity and decreased levels of anti-inflammatory cytokines in visceral fat obesity [151,152]. Patients with diabetes often exhibit elevated levels of TNFα in their plasma and muscles [153,154,155]. TNFα negatively affects insulin signaling by attenuating the insulin-stimulated tyrosine phosphorylation of the insulin receptor and insulin receptor substrate 1 (IRS1) in the WAT and muscles, leading to insulin resistance [153,154,155,156]. Increased levels of TNFα can stimulate fatty acid uptake in the liver, contributing to fat accumulation and the production of ROS [157,158]. Moreover, TNFα promotes the incorporation of fatty acids into diacylglycerol (DAG), suggesting its role in inducing insulin resistance in skeletal muscle [156]. In summary, TNFα is an inflammatory cytokine produced by monocytes and macrophages, and it is involved in the development of insulin resistance by impairing insulin signaling in the WAT and muscles, with visceral fat obesity being a significant site of its production in individuals with obesity. TNFα also influences hepatic fatty acid uptake, leading to fat accumulation and the production of ROS in the liver. Interestingly, its effects on insulin resistance extend to skeletal muscles.

### 4.4. IL-6

Interleukin-6 (IL-6) exhibits both pro-inflammatory effects as an adipokine and anti-inflammatory effects as a myokine [126,159,160]. The dual functions of IL-6 in different organs can be attributed to different inducers and signaling pathways that stimulate its expression. As a myokine, IL-6 is primarily secreted by skeletal muscles in response to exercise. Muscle-derived IL-6 regulates glucose and lipid metabolism by enhancing the insulin signaling pathway [159]. Conversely, elevated levels of circulating IL-6 have been observed in individuals with T2D, obesity, and insulin resistance [161,162]. As an adipokine, IL-6 has been positively correlated with BMI, with approximately one-third of circulating IL-6 originating from the adipose tissue. Notably, visceral adipose tissue is a significant source of IL-6 in relation to obesity [163,164,165]. In the adipose tissue, IL-6 expression is predominantly produced by macrophages, with its expression being induced by the activation of the NF-κB signaling pathway [163]. Furthermore, IL-6 hampers insulin signaling and reduces insulin-dependent glucose uptake by inhibiting the expression of GLUT4 and IRS1 in adipocytes [166,167]. In summary, IL-6 exerts distinct roles as an adipokine and myokine. Its myokine activity contributes to metabolic improvements, whereas its adipokine activity, particularly in the visceral adipose tissue, is associated with insulin resistance and metabolic disorders. However, further investigations are required to elucidate the underlying mechanisms and potential therapeutic implications of IL-6.

### 4.5. RBP4

Retinol binding protein 4 (RBP4) is primariily secreted by hepatocytes and functions as a carrier for transporting retinol from the liver to peripheral tissues [168]. However, it has also been identified as an adipokine secreted by adipocytes and macrophages [169,170].The levels of circulating serum RBP4 are elevated under insulin-resistant conditions. Visceral adipose tissue is a major source of increased levels of serum RBP4, which have been associated with a higher BMI [171,172]. Moreover, elevated levels of serum RBP4 have been linked to adverse health effects including increased blood pressure and plasma levels of cholesterol and triglycerides [171,173]. Consequently, RBP4 is considered as a marker of intra-abdominal fat accumulation and inflammation associated with obesity. Adipocyte-derived RBP4 acts in an autocrine or paracrine manner to inhibit the insulin-induced phosphorylation of IRS1 [170,174]. Studies showed that adipocyte-specific *Glut4* knockout mice exhibited increased expression levels of RBP4 in the WAT, contributing to glucose intolerance and insulin resistance [170,171].

## 5. Organokines and Dietary Interventions

### 5.1. Caloric Restriction

Caloric restriction (CR) is defined as reducing calorie intake below energy demands without eliminating of essential nutrients [175,176,177]. CR has emerged as a popular approach to treating T2D and obesity. Extensive studies have reported the effectiveness of CR in regulating organokines (hepatokines, myokines, and adipokines), thereby ameliorating the pathophysiology of obesity and T2D. In patients with T2D, CR intervention for 12 weeks significantly down-regulated circulating fetuin-A concentrations, resulting in improved blood pressure, plasma glucose, visceral fat, and lipid profiles [178]. FGF21 is a fasting-induced hepatokine that is gaining attention as a metabolic regulator [52]. A methionine-restricted diet was shown to increase hepatic FGF21 [179]. In addition, in a preclinical study, Fgf21^-/-^ mice exhibited increased high-fat (HF) diet-induced inflammation in the WAT and the liver compared with that in wild-type (WT) mice, while a methionine-restricted diet reduced inflammation in an FGF21-dependent manner [180]. This suggests that the methionine-restricted diet restored endogenous FGF21 and protected against HF diet-induced inflammation in the WAT and liver. Likewise, it was demonstrated that a leucine-deprived diet markedly reduced body weight and induced browning in WAT by increasing hepatic FGF21 gene expression in mice [181]. Despite these beneficial effects of methionine and leucine-restricted diets, prolonging one essential amino acid-deficient diet can jeopardize the animal’s health [182,183]. Methionine has been reported to be crucial for normal metabolic processes [184] and immunity, such as T-cell activation and differentiation [185,186]. Recent studies showed that methionine restriction aggravated tumor progression by repressing T-cell activation [187] and impaired the protein synthesis of translation-initiation machinery and antioxidant enzymes in mice [188]. Furthermore, long-term leucine deprivation led to dramatic weight loss, dysregulation of energy homeostasis, and increased prenatal and neonatal mortalities in mice [182,183]. These studies suggest the importance of seeking the optimal amount of dietary amino acids and an experimental period that can faithfully reproduce the beneficial metabolic effects of methionine and leucine-restricted diets. It is also necessary to consider other factors, such as disease state, to drive the therapeutic benefits of methionine and leucine-restricted diets.

Moreover, information from nutritional studies has indicated that CR can improve organokines in relation to inflammation. Other studies showed that circulating levels of amyloid A protein, interferon-gamma (IFN-γ), interleukin-6 (IL-6), tumor necrosis factor alpha (TNF-α), and C-reactive protein (CRP) were prominently reduced in patients with obesity by CR [189,190]. Notably, a randomized controlled trial of CR showed that CR significantly enhanced T-cell proliferation (TP) and prostaglandin E2 production [191] while reducing serum CRP levels [192] compared with the levels before CR. These results indicate that CR potentially affects pro-inflammation marker reduction and enhances innate immunity. There are several underlying molecular mechanisms by which CR reduces the inflammatory response. First, CR increases adiponectin, which may affect down-regulates phosphatidylinositol 3-kinase (PI3K)/NF-κB pathways and inhibit the NLRP3 inflammasome [193,194,195]. A CR-mediated increase in adiponectin can also inhibit macrophage differentiation and induce macrophage polarization from the M1 to M2 state [196,197,198]. Moreover, CR increases circulating ketone bodies such as β-hydroxybutyrate (BHB), which is known to block NLRP3 inflammasome-mediated inflammatory responses [199,200].

These studies show that CR, including amino acid restriction, is an appealing approach for combating obesity and T2D. However, there are some limitations. The findings from preclinical studies may be less applicable to humans. For example, the methionine-restricted diets used in preclinical studies included 0.17% methionine. Animal studies showed high adherence to this diet, while clinical studies showed high withdrawal rates due to poor palatability [201]. This review considered the positive effects of CR on improving metabolic profiles. However, several studies produced conflicting results that CR negatively impacts bone health, wound healing, and immune responses [202]. Future studies are warranted to investigate the effects of caloric restriction and various feeding regimens on obesity and T2D.

### 5.2. Dietary Fiber

Dietary fiber consists of highly complex substances such as nondigestible carbohydrates and lignin that cannot be digested in the upper gut [203]. For example, whole-grain, vegetables, legumes, and fruits contain different types of dietary fiber [203]. Accumulating evidence showed that dietary fiber consumption is associated with a low risk of obesity and T2D [203,204]. The fermentation of dietary fiber by the gut microbiome produces high amounts of microbial metabolites, including short-chain fatty acids (SCFAs), succinate, lactate, and branched-chain fatty acids (BCFAs) [205,206]. Several studies demonstrated a link between SCFAs intake and improvements in metabolic phenotypes [207,208]. In subjects with obesity, consumption of vinegar containing 1.5 g of SCFAs (acetic acid), led to a significant decrease in BMI, body weight, waist circumference, and serum triglycerides, demonstrating the role of SCFAs in body weight control [207]. Another intervention study showed that inulin propionate ester intake over 24 weeks significantly decreased weight gain and prevented deterioration in insulin sensitivity in adults who were overweight [208]. Notably, SCFAs have been reported to stimulate the adipose-tissue-derived satiety hormone leptin in mice [209] and humans [210]. The obesity insulin-resistant state is intimately related to chronic inflammation in adipose tissue. Treatment with butyrate, one of the SCFAs, markedly inhibited secretion of pro-inflammatory cytokines, such as IL-6, TNF-α, and MCP-1 in the co-incubation of murine 3T3-L1 adipocytes and RAW264.7 macrophages [211]. Furthermore, propionate treatment of adipose tissue explants obtained from patients who were overweight significantly downregulated inflammatory cytokines such as CCL5 and TNF-α [212]. These results indicate the beneficial role of SCFAs, obtained by fiber intake in preventing obesity and T2D.

### 5.3. ω3 Polyunsaturated Fatty Acids (PUFAs)

A diet enriched in ω3 polyunsaturated fatty acids (PUFAs) is known to exert beneficial effects on metabolic health in humans. Studies in rodents revealed that ω3 PUFAs contributed to obesity phenotype improvements, including WAT inflammation, insulin sensitivity, glucose tolerance, and colonic inflammation, by targeting gut microbiota [213,214]. Moreover, ω3 PUFA supplementation reportedly improves dyslipidemia and hyperglycaemia [215]. Recent evidence suggests that PUFAs supplementation targets adipose tissues; PUFAs enhanced brown adipose tissue recruitment and WAT browning by increasing uncoupling protein-1 levels and mitochondrial oxidative capacity [216,217,218,219]. Notably, ω3 PUFAs are known to affect several organokines. A recent study showed that ω3 PUFA supplementation (1250 mg thrice/day) markedly increased serum irisin levels in patients with T2D [220]. ω3 PUFA supplementation also led to a reduction in FBS and HbA1C in these patients [220]. In mice, ω3 PUFAs markedly increased FGF21 secretion from brown or beige adipocytes, thereby inducing brown and beige differentiation via GFP120 activation [221]. ω3 PUFAs strongly inhibit inflammatory cytokine secretion in the adipose tissue. In addition, treatment with ω3 PUFAs (DHA and EPA) effectively inhibited inflammatory signaling pathways and improved insulin sensitivity, potentially through GPR120, in obese mice [222].

### 5.4. Selenium

Selenium is an essential micronutrient for human health [223] and is a crucial constituent in selenoproteins, which have diverse biological functions, including anti-inflammation and antioxidation [223]. Selenoproteins P plays an important role in regulating T2D. A study utilizing selenoprotein P-neutralizing antibodies demonstrated that glucose metabolism is significantly improved in high-energy diet-induced mice [224]. However, low or high selenium supplementation increases insulin resistance by up- or down-regulating selenoproteins in our body, suggesting that moderate intake of selenium is crucial [223,225]. Furthermore, selenium supplementation dramatically improved plasma levels of IGF-1, FGF-21, adiponectin, and leptin levels, protecting against diet-induced obesity in mice [226].

### 5.5. Vitamins

#### 5.5.1. Vitamin D

Vitamin D insufficiency results from inadequate vitamin D intake, high vitamin D catabolism, inadequate exposure to sunlight, and inefficient production in the skin [227]. Vitamin D insufficiency plays a significant role in the pathogenesis of a wide range of metabolic diseases such as T2D and obesity [228,229]. Specifically, vitamin D has been demonstrated to enhance insulin release and decrease insulin resistance in T2D [230]. Vitamin D supplementation has been shown to increase muscle irisin levels and FDNC5 gene expression with increasing serum vitamin D levels in the streptozotocin-diabetic rats [231]. In addition, a recent clinical study showed that 6 months of vitamin D supplementation increased serum irisin levels [232]. The anti-inflammatory effects of vitamin D in various diseases are well-reported [233,234,235,236,237]. For example, 1,25(OH)_2_D_3_ markedly inhibits IL-6, leptin, and nuclear factor-κB in human adipocytes [238]. Furthermore, 25-hydroxyvitamin D3 [(25(OH)D_3_)], but not vitamin D3, effectively suppressed adipokine expression in human adipose tissues [238]. Notably, vitamin D receptor (VDR) deletion from human adipose tissue up-regulated inflammatory signaling activity, suggesting that the anti-inflammatory effects of vitamin D on adipose tissues are mediated by VDR [238]. These results suggest that vitamin D supplementation may improve obesity-associated metabolic complications by inhibiting inflammation in the adipose tissues.

#### 5.5.2. Vitamin A

Retinoic acid, the carboxylic acid form of vitamin A (retinol), has beneficial effects on energy metabolism [239,240]. All-trans retinoic acid (ATRA) is known to modulate gene expressions via retinoid X receptors (RXRs) and the retinoic acid receptors (RARs). Several studies reported that ATRA supplementation reduced leptin expression in the adipose tissues [241], and inhibited body weight gain and adiposity [242]. ATRA regulates the secretion of signaling proteins from adipose tissues, such as leptin and retinol-binding protein 4 (RBP4), to maintain energy balance and insulin sensitivity [243,244,245,246]. Furthermore, ATRA treatment of C2C12 myoblasts increases irisin secretion in a dose-dependent manner [247]. β-Carotene, known as a provitamin A carotenoid, inhibits oxidative stress-mediated inflammation by increasing the secretion of adiponectin in 3T3-L1 preadipocytes, highlighting its role in remodeling of oxidative stress-mediated dysregulated adipokines [248]. Lycopene, a non-provitamin A carotenoid typically found in tomatoes and tomato products, suppresses pro-inflammatory markers in the WAT of rodents and humans [249]. Notably, apo-10′-lycopenoic acid, a metabolite of lycopene, possesses anti-inflammatory effects in WAT via RAR [250]. A recent study utilizing non-target metabolite analysis of tomato revealed that β-carotene and lycopene improved the adiponectin signaling pathway in C2C12 myotubes [251]. Carotenoids, including lycopene and β-carotene, are prominently stored in the WAT [252].This dominant carotenoid and lycopene accumulation in the adipose tissue, followed by diet intervention, can explain the strong anti-inflammatory effects of carotenoids in WAT.

In vitro studies have limitations because of the lack of an in vivo microenvironment and various factors that enable cell communication. For example, cultured myotubes exhibit lower insulin-stimulated glucose uptake compared with in vivo systems [253]. Future studies should consider multiple factors in testing a therapeutically beneficial diet/nutrition to prevent and treat obesity and T2D.

#### 5.5.3. Vitamin B12 and Folate

Vitamin B12 and folate are crucial cofactors for transforming homocysteine to methionine [254,255]. Vitamin B12 deficiency is highly prevalent among patients with T2D [256]. Mounting evidence revealed that vitamin B12 and folate supplementation improved obesity and insulin sensitivity in T2D [257]. Vitamin B12 and folate deficiency can reportedly disrupt adipokine expression [258,259], possibly leading to an increased risk of obesity and cardiovascular diseases. An animal study showed that vitamin B12 and folic acid treatment increased adiponectin and decreased leptin concentration [260]. Furthermore, a recent study showed that early supplementation with vitamin B12 and folic acid improved leptin concentration and the leptin-adiponectin ratio, suggesting the possibility of increased insulin sensitivity [261].

## 6. Exercise

Physical exercise offers benefits beyond simply increasing energy expenditure, as it also has the ability to reshape overall energy metabolism in the body. The positive effects of engaging in physical activity are widely recognized when it comes to addressing metabolic disorders and their associated complications. These conditions include obesity, metabolic syndrome, cardiovascular diseases, and non-alcoholic fatty liver disease (NAFLD) [262,263]. A group of peptides and proteins released by muscles, collectively known as myokines, play a significant role in the health benefits associated with exercise. Recent investigations into muscle secretomes have unveiled that both aerobic exercise and strength training stimulate the release of numerous myokines [264]. Notably, it is crucial to recognize that the production of “beneficial” myokines is not only promoted by physical exercise but also suppressed by physical inactivity, emphasizing the importance of lifestyle choices in promoting a healthy lifespan [264,265,266]. The mechanisms underlying the impact of physical activity have been thoroughly studied, and recent research has further expanded our understanding [267]. In the context of physical exercise, skeletal muscle plays a crucial role in closely interacting with adipose tissue, pancreas, and liver. This interaction is essential for meeting the energy requirements of physical activity and facilitating favorable effects on overall energy metabolism throughout the body [268,269].

### 6.1. Acute Exercise

“Acute exercise” can be defined as a single instance of exercise, including endurance activities (aerobic exercise) and resistance activities (strength training or weightlifting) [270]. In a study involving insulin-resistant individuals, the impact of a single 45 min exercise session on de novo lipogenesis was examined in young, lean, insulin-resistant individuals [271]. Participants’ intrahepatic lipid content was measured before and after the session. Afterward, they were provided a high-carbohydrate liquid meal, and de novo lipogenesis rates were determined using deuterium-labeled water. The findings indicated that de novo lipogenesis activity was significantly lower in the exercise group compared with the resting group. This reduction in de novo lipogenesis prevented the increase in intrahepatic lipid content observed in the resting condition, suggesting that exercise positively influenced de novo lipogenesis rates following the meal. Although plasma glucose and insulin levels were similar between the exercise and resting conditions, acute exercise led to a threefold increase in postprandial muscle glycogen synthesis. By redirecting ingested carbohydrates away from the liver and toward skeletal muscle, exercise reduces hepatic de novo lipogenesis and the synthesis of triglycerides in the liver. This study provides compelling evidence supporting the notion that insulin resistance in muscles plays a role in the early development of atherogenic dyslipidemia and non-alcoholic fatty liver disease (NAFLD).

One of the well-studied hepatokines related to acute exercise effect is FGF21. Following acute endurance exercise, the serum concentration of FGF21 increases gradually [272,273,274,275]. Immediate or slight changes in FGF21 levels are typically observed immediately after exercise, with peak values occurring around one hour post-exercise [275]. Limited studies have investigated FGF21 levels beyond this time frame, but it appears that FGF21 concentration tends to return to normal relatively quickly during the resting period. A recent study demonstrated that the increase in plasma FGF21 levels induced by exercise was absent in T2D patients. This suggests that the production of FGF21 in response to acute exercise is altered in individuals with metabolic disturbances. Although T2D patients had higher baseline FGF21 levels compared with healthy individuals, it appears that hyperinsulinemia or hepatic insulin resistance hampers the exercise-induced secretion of FGF21 [276]. Previous research has also indicated that obese individuals with hyperinsulinemia have lower FGF21 secretion compared with healthy individuals. In summary, impaired exercise-induced FGF21 secretion may be associated with factors such as hyperinsulinemia or hepatic insulin resistance in individuals with metabolic disruptions [277].

Different studies related to hepatokines and acute exercise evaluated that following a single bout of exercise (at 60–70% of VO2 max, burning 500 kcal), obese individuals experienced an immediate rise in serum phosphofetuin-A (Ser312) levels, which returned to baseline within 24 h. Future studies need to take into account multiple factors to produce a therapeutically beneficial diet/nutrition for the prevention and treatment of obesity and T2D. Notably, glucose and insulin levels during an oral glucose tolerance test (OGTT) were significantly reduced 24 h after the exercise session [278]. This evidence suggests that the exercise-induced decrease in fetuin-A levels may contribute to the acute health benefits of exercise observed in this context [279].

For the myokines, research examining the relationship between exercise and follistatin (FST) consistently demonstrates an increase in FST levels following exercise. The majority of studies investigating this relationship focus on acute effects rather than long-term effects. Various types of exercise, including resistance [280,281,282], endurance [273,282,283], and high-intensity interval training (HIIT) [272,282], have been found to raise plasma/serum FST levels after an acute exercise session, with increases ranging from approximately 5% to 500%. The highest response has been observed in younger individuals performing exercise at higher intensities. However, factors such as protocol variations may limit the increase in FST levels in other studies. The concentration of FST typically peaks around 3–4 h after exercise and then gradually decreases, although in some studies, elevated concentrations have been observed for up to 72 h post-exercise [284].

While an increase in serum IL-15, another myokine, was not associated with enhanced muscle protein synthesis in some studies [285,286], other studies have reported an elevation of IL-15 following resistance exercise [272,287]. Acute resistance exercise has been found to stimulate the production of IL-15. The increase in IL-15 typically occurs within the first hour of recovery and is unaffected by the availability of carbohydrates or fat prior to exercise. However, there is some debate regarding the role of IL-15 in exercise-induced muscle hypertrophy [272]. The discrepancy in findings may be attributed to variations in the type and intensity of exercise, as well as the fitness level of the participants [288,289]. It has been observed that individuals with higher fitness levels, including resistance and endurance-trained athletes, tend to have higher baseline levels of IL-15 compared with untrained individuals [289]. Interestingly, an acute bout of high-intensity interval endurance training did not result in changes in IL-15 concentration in sedentary subjects [272].

In terms of lipid oxidation related to adiponectin, a single 1 h bout resistance exercise enhances postprandial lipid oxidation throughout the entire body in obese men with prediabetes [290]. Resistance exercise was found to enhance several metabolic processes in the body. These included increased whole-body lipid oxidation, enhanced skeletal muscle mitochondrial respiration, upregulated expression of oxidative genes in skeletal muscle, and an increase in the expression of important lipolysis genes in adipose tissue [290]. Furthermore, a single session of acute exercise, regardless of intensity, leads to a notable increase in plasma adiponectin levels in sedentary, abdominally obese men. Additionally, high-intensity exercise results in a significant reduction in plasma triglyceride levels in the same individuals, while low-intensity exercise does not produce this effect. Importantly, the elevated adiponectin levels persist for up to 72 h after completing three exercise sessions within one week, without any change in weight or body composition. These findings suggest that even a single session of aerobic exercise can lead to positive changes in adiponectin levels, highlighting another potential health benefit associated with exercise [291]. This upregulation of adiponectin expression may contribute to the observed reduction in insulin concentration and the elevation of whole-body lipid oxidation following resistance exercise [290,292].

The study presented valuable insights into the effects of acute exercise on organokines and metabolic responses, shedding light on their potential roles in health modulation. However, certain limitations warrant consideration. First, the study primarily focused on young, lean, and insulin-resistant individuals, potentially limiting the generalizability of the findings to broader population groups. Moreover, while the study identified associations between exercise, organokine secretion, and metabolic changes, causality could not be definitively established due to the observational nature of the research. Lastly, the intricate interplay of genetic factors, lifestyle, and other environmental elements that influence organokine responses was not extensively addressed, indicating the need for more comprehensive studies in more diverse populations and conditions.

### 6.2. Chronic Exercise

“Chronic exercise”, often called endurance training or aerobic exercise, involves engaging in regular and repeated sessions of physical activity sessions over an extended period [270]. This type of exercise is characterized by moderate-intensity activities performed consistently to improve cardiovascular fitness, increase endurance, and support overall health [293,294]. Chronic exercise includes activities such as running, cycling, swimming, brisk walking, and participating in aerobic classes [295].

Chronic exercise training is likely associated with a decrease in circulating irisin, considering the following possibilities. Irisin has been found to be released not only from muscles but also from adipose tissue, making it an adipokine as well as a myokine [296,297]. Studies have shown that circulating irisin levels were accompanied by concurrent weight and fat loss following surgery and chronic exercise [298,299,300]. These studies indicate that long-term exercise training potentiates irisin reduction by weight and fat loss. Furthermore, cross-sectional studies have demonstrated that higher circulating irisin levels are positively associated with insulin resistance and fasting blood glucose in non-diabetic individuals, indicating a potential role of irisin in the regulation of glucose homeostasis [301,302]. It is hypothesized that irisin is released in greater amounts by adipose and muscle tissues as a compensatory response to overcome irisin resistance and counteract impaired insulin function [301,303]. Given that chronic exercise training improves insulin resistance, it is logical to assume that circulating irisin levels would diminish as a consequence of exercise training. These findings may be explained by the reduction in metabolic stress and inflammation that occurs as a result of exercise-induced fat loss, rather than solely being attributed to the exercise itself. Additionally, some studies included in the analysis did not measure irisin levels immediately after exercise sessions, which would be helpful in future research, considering its proposed short lifespan in the bloodstream [304].

The impact of chronic exercise on circulating levels of FGF21 in individuals with metabolic disorders has been investigated in various studies, and the results appear to be conflicting. The contradictory findings regarding the effects of exercise on circulating FGF21 suggest that both muscle and liver respond to the elevation of FGF21 induced by exercise, but the dominant response may vary under different conditions. Some studies support the notion that chronic exercise, either alone or in combination with dietary intervention, can significantly reduce circulating FGF21 levels in obese or elderly individuals [305,306,307]. However, other studies did not observe any effect of chronic exercise on FGF21 levels in obese or diabetic patients [308,309]. It is important to consider that these discrepancies could be attributed to methodological issues. Firstly, FGF21 levels are influenced by various factors such as fasting status [310], nutrient intake [311], and circadian rhythm control [311,312], which were not always specified in these studies. Additionally, it is important to consider that the response of FGF21 to exercise is temporary and influenced by the timing of sample collection. This can explain why there was no significant difference observed between FGF21 levels before exercise and 48 h after exercise [313]. Furthermore, not all the studies examined changes in systemic levels of insulin or free fatty acids, hepatic fat content, or cardiorespiratory fitness, all of which have been shown to impact FGF21 levels.

Furthermore, exercise influences the levels of adipokines by modifying the expression of genes and activating or inactivating proteins that are part of their signaling pathways [159]. It appears that chronic exercise is necessary to restore the physiological effects of leptin. Meta-analyses have shown that long-term aerobic, resistance, and combined exercise lead to reductions in fat mass accompanied by lower levels of leptin [314,315]. Another previous study found that chronic exercise training for at least two weeks, whether aerobic or resistance training, resulted in decreased leptin levels in elderly postmenopausal women [314]. This reduction in leptin levels was dependent on the percentage of body fat, regardless of age and sex.

The mechanisms involved in this decrease during moderate to severe resistance training include factors such as peripheral glucose uptake, sympathetic stimulation of the adrenal gland, lactate and acidosis, and glycogen depletion specifically in elderly postmenopausal women [316]. This improvement in leptin sensitivity can be attributed to decreased feedback inhibitors on leptin receptors (LepR), the creation of an anti-inflammatory environment throughout the body induced by exercise, and reduced oxidative stress in the hypothalamus [159,315,317,318]. In addition to its traditional role in increasing sympathetic nervous system activity to promote overall energy expenditure, restored leptin sensitivity in peripheral organs can also facilitate the maintenance of reduced body fat. Leptin has been found to increase glucose and free fatty acid uptake and oxidation in skeletal muscle, while decreasing intrahepatic lipid content by promoting fatty acid oxidation [319]. Therefore, leptin may play a role in redirecting nutrients away from white adipose tissue, aligning with its function as an “adipostat” that tightly regulates adipocyte size under normal physiological conditions.

However, as previously discussed, the regulation of organokines appears to be governed by a spectrum of variables beyond mere training intensity and duration. Factors such as nutrition, lifestyle, circadian rhythms, and an array of other influences seemingly contribute to the intricate tapestry of organokine production [320,321]. Hence, it becomes imperative to comprehensively explore the multi-faceted landscape of exercise-induced cellular metabolism in a comprehensive manner. In this pursuit, particular emphasis should be placed on unraveling the impact of multi-factors, including nutrition, lifestyle, and circadian rhythms on cellular metabolism.

## 7. Conclusions

**Figure 1:**
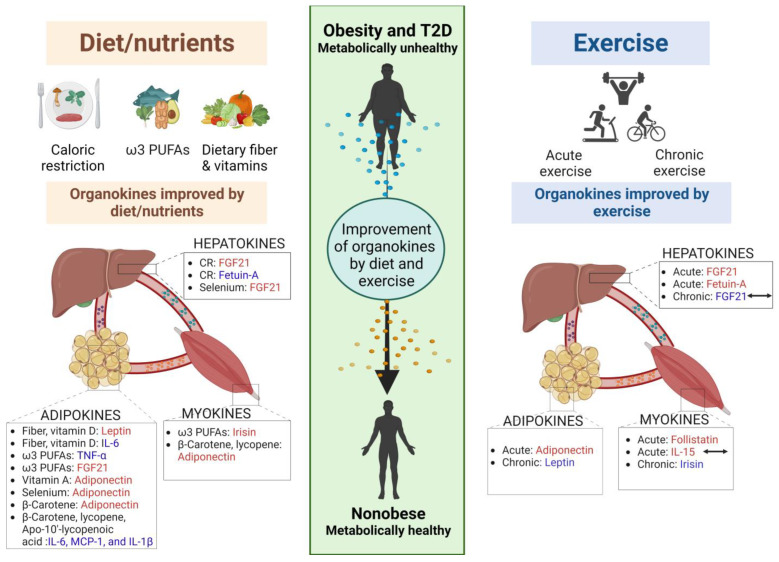
The role of diet and exercise in remodeling of organokines in obesity and T2D. The liver, muscle, and adipose tissue are potent endocrine organs that secrete hepatokines, myokines, and adipokines. These organokines exert endocrine or paracrine actions and interact with each other. The changing profile of organokines in obesity and T2D is ameliorated by diet/nutrients and exercise, thereby improving the pathophysiology of obesity and T2D. Red organokines represent up-regulation, blue organokines indicate down-regulation, and double-sided arrows indicate controversial changes by exercise. CR: caloric restriction; acute exercise: a single instance of exercise; chronic exercise; regular and repeated sessions of physical activity; FGF21: fibroblast growth factor 21; IL: interleukin; TNF: tumor necrosis factor; CCL: C-C Motif Chemokine Ligand; PUFA: polyunsaturated fatty acids; T2D: type 2 diabetes; MCP-1: monocyte chemoattractant protein-1. Figure created using BioRender.com.

In this review, we summarized the organokines that play crucial roles in the pathophysiology of obesity and T2D. CR has emerged as an effective approach for regulating organokines, leading to improvements in glucose metabolism, insulin sensitivity, and lipid profiles. Dietary interventions, such as fiber consumption and supplementation with ω3 PUFAs, selenium, and vitamins, have shown promising effects in modulating organokines and mitigating inflammation in adipose tissues (Figure 1 and Table 1). Furthermore, exercise has been identified as a powerful tool for reshaping energy metabolism and influencing the secretion of myokines (Figure 1 and Table 2). These findings highlighted the potential of targeting organokines through lifestyle interventions as a valuable strategy for the management and prevention of obesity and T2D. Overall, understanding the complex interactions among organokines, metabolic disorders, and lifestyle factors provides valuable insights into the development of effective therapeutic approaches.

Unlike many previous publications focusing solely on the role of organokines in metabolic disorders, our review is unique in that it explored the multifaceted influence of lifestyle interventions on these signaling molecules. By summarizing the effects of nutrition and exercise on organokine regulation, we provided a comprehensive perspective that extends beyond the conventional examination of organokines in isolation. However, in addition to making important contributions, our review paper has certain limitations that warrant consideration. Specifically, the complex interactions between organokines, metabolic disorders, and lifestyle factors may have been confounded by other variables that were not fully addressed in the studies analyzed. Inheritance of genetic predisposition, environmental influences, and variations in individual responses to interventions can all contribute to a complex interaction of outcomes. Furthermore, this review did not include organokines produced by the brain or brown adipose tissues. Recently, the function of cytokines produced in the central nervous system by astrocytes, neurons, and microglia has been gaining attention [322]. Future studies are needed to elucidate the effects of organokines produced in the brain and brown adipose tissues and their modulation by diet and exercise.

In conclusion, while targeting organokines holds promise for the management of obesity and T2D, limitations, such as an incomplete understanding of the underlying mechanisms, short-term focus of studies, individual variations, complex interplay with other factors, and challenges in real-world implementation need to be addressed.

## 8. Future Directions

Despite the potential of targeting organokines for the management of obesity and T2D, this review had several limitations. First, the exact mechanisms underlying the regulation and interactions of organokines in metabolic disorders are not fully understood. The complexity of signaling pathways and crosstalk between different organs and tissues makes it challenging to pinpoint specific cause-and-effect relationships. Additionally, individual variations in the expression of organokines and responses to lifestyle interventions further complicate the interpretation of results and the development of standardized treatment approaches. Most studies investigating the effects of CR, dietary interventions, and exercise on organokines have focused on short-term interventions or exercise bouts. Hence, long-term adherence to these interventions and their sustained effects on organokine profiles and metabolic outcomes remain relatively unexplored.

In addition, most studies investigated the solitary effects of the intervention on organokines in obesity and T2D. Future studies should address the synergistic effects of diet/nutrients and exercise on organokine reorganization and its impact on the pathophysiology of obesity and T2D. Furthermore, most studies have been small-scale or conducted using animal models, limiting the adaptability and generalizability of the findings to larger populations. Other factors, such as genetic predisposition, gut microbiota composition, and environmental influences, can also significantly affect the regulation and functionality of organokines. Therefore, further research is required to better understand these interactions and develop comprehensive therapeutic strategies that target multiple aspects of metabolic diseases.

The idea of inheritance in organokines refers to how genetic factors can affect the creation, control, and impact of these specific signaling molecules, which were produced by different body organs. Although the study of organokines is relatively new, the connection between genetics and the function organokines is a complex area that is gaining attention. Genetic variations can affect organokine production, increasing individual’s susceptibility to metabolic diseases, such as obesity and T2D [323,324]. For example, the genetic variants in the TNF-α or IL-6 are more frequently found in obese children compared with nonobese children [15,320,321]. Increasing evidence has shown that maintaining the optimal expression pattern of organokines (e.g., leptin) in parents is another crucial factor in determining the health of future generations [325].

It has been demonstrated that parental lifestyle and environmental exposures can affect epigenetic modifications of somatic and germline cells, delivering a unique epigenetic landscape to offspring [325,326]. For example, it has been found that maternal supplementation with n-6 and n-3 PUFAs during pregnancy modulates the epigenome, leading to a significant reduction in leptin levels in young and adult offspring [327,328,329]. However, more studies are needed to elucidate the role of organokines in the transgenerational inheritance of metabolic diseases.

Finally, although lifestyle interventions can effectively modulate the expression of organokines and improve metabolic health, their implementation and long-term adherence to real-world setting is challenging. Factors such as socio-economic status, access to healthy foods, and barriers to physical activity can affect the feasibility and sustainability of lifestyle interventions, limiting their widespread application and effectiveness. Future studies should aim to overcome these limitations and provide comprehensive insights into the role of organokines in metabolic disorders, leading to the development of effective and personalized therapeutic strategies.

## Figures and Tables

**Table 1:** Modulation of organokines by dietary intervention.

Nutrient/Diet	Organokine	Name	Effect and Biological Action	References
Calorie restriction	Hepatokine	Fetuin-A	Calorie-restriction intervention for 12 weeks decreased circulating fetuin-A concentrations, with improved blood pressure, plasma glucose, visceral fat, and lipid profiles.	[178]
Methionine-restricted diet	Hepatokine	FGF21	Methionine-restricted diet increased hepatic FGF21.	[179]
Leucine-restricted diet	Hepatokine	FGF21	Leucine-restricted diet markedly reduced body weight and induced browning in subcutaneous WAT by increasing hepatic FGF21 gene expression in mice.	[181]
Dietary fiber (SCFAs)	Adipokine	Leptin	SCFAs stimulated the secretion of the satiety hormone leptin from adipose tissues.	[209,210]
	Adipokine	IL-6 and TNF-α	Butyrate treatment suppressed pro-inflammatory cytokine production, including IL-6, TNF-α, and MCP-1 in the co-incubation of murine 3T3-L1 adipocytes and RAW 264.7 macrophages.	[211]
	Adipokine	CCL5 and TNF-α	Propionate treatment of adipose tissue explants obtained from patients who were overweight significantly downregulated inflammatory cytokines such as CCL5 and TNF-α.	[212]
ω3 PUFAs	Myokine	Irisin	ω3 PUFAs supplementation (1250 mg thrice/day) increased serum irisin levels in patients with T2D.	[220]
	Adipokine	TNF-α	ω-3 PUFAs supplementation inhibited inflammatory cytokine expression in the adipose tissues of obese mice.	[222]
	Adipokine	FGF21	n-3 PUFAs up-regulated FGF21 expression and secretion in brown and beige adipocytes, thereby inducing brown and beige differentiation.	[221]
Selenium	Adipokine Hepatokine	FGF21 Leptin Adiponectin	Selenium supplementation improved the profile of plasma levels of FGF-21, adiponectin, and leptin levels, reducing diet-induced adiposity in mice.	[226]
Vitamin D	Adipokine	Leptin	1,25(OH)_2_D_3_ decreased the secretion of leptin in human adipocytes.	[238]
	Adipokine	IL-6 and nuclear factor-Κb	1,25(OH)_2_D_3_ inhibited IL-6 and nuclear factor-Κb in human adipocytes.	[238]
Vitamin A	Adipokine	Leptin	All-trans retinoic acid decreased leptin expression in the adipose tissues.	[241]
	Adipokine	Leptin and RBP4	All-trans retinoic acid regulated the secretion of leptin and RBP4 from adipose tissues.	[243,244,245,246]
	Myokine	Irisin	All-trans retinoic acid treatment of C2C12 myoblasts increased myokine irisin secretion in a dose-dependent manner.	[247]
β-Carotene (pro-vitamin A carotenoid)	Adipokine	Adiponectin Inflammatory cytokines	β-Carotene treatment significantly decreased inflammation in 3T3-L1 adipocytes, while increasing the secretion of adiponectin.	[248]
Lycopene (non-provitamin A carotenoid)	Adipokine	IL-6, MCP-1, and IL-1β	Lycopene inhibited pro-inflammatory markers in the WAT of rodents and humans.	[249]
Apo-10′-lycopenoic acid	Adipokine	IL-6 and IL-1β	Apo-10′-lycopenoic acid exerts anti-inflammatory effects in WAT via RAR.	[250]
β-Carotene and lycopene	Myokine	Adiponectin	Non-target metabolite analysis of tomato demonstrated that β-carotene and lycopene enhanced the adiponectin signaling pathway in C2C12 myotubes.	[251]
Vitamin B12 and folate	Adipokine	Adiponectin and leptin	Vitamin B12 and folic acid treatment improved adiponectin and leptin profiles in mice and humans.	[260,261]

FGF21: Fibroblast growth factor 21; WAT: white adipose tissue; SCFAs: Short-chain fatty acids; IL: interleukin; TNF: Tumor necrosis factor; CCL: C-C Motif Chemokine Ligand; PUFA: polyunsaturated fatty acids; T2D: type 2 diabetes; RBP: Retinol binding protein; MCP-1: monocyte chemoattractant protein-1; RAR: retinoic acid receptors.

**Table 2:** Modulation of organokines by exercise.

Exercise	Organokine	Name	Effect and Biological Action	References
Acute	Myokine	Follistatin (FST)	Acute exercises, including resistance, endurance, and HIIT, raise plasma/serum FST levels ranging from approximately 5% to 500%.	resistance [280,281,282], endurance [273,282,283], HIIT [272,282,284]
			The concentration of FST typically peaks around 3-4 h after exercise and then gradually decreases, although in some studies, elevated concentrations have been observed for up to 72 h post-exercise.	[284]
	Myokine	IL-15	Resistance exercise elevates IL-15 within the first hour of recovery and is unaffected by the availability of carbohydrates or fat prior to exercise.	[272,287]
			Individuals with higher fitness levels, including resistance and endurance-trained athletes, tend to have higher baseline levels of IL-15 compared with untrained individuals.	[289]
			An acute bout of high-intensity interval endurance training did not result in changes in IL-15 concentration in sedentary subjects.	[272]
	Myokine Hepatokine	FGF21	Immediate or slight changes in FGF21 levels are typically observed immediately after acute endurance exercise, with peak values occurring around 1 h post-exercise.	[272,273,274,275]
			T2D patients, despite having higher baseline FGF21 levels compared with healthy individuals, hyperinsulinemia or hepatic insulin resistance can hinder the exercise-induced secretion of FGF21.	[276]
			Obese individuals with hyperinsulinemia have lower FGF21 secretion compared with healthy individuals.	[277]
	Hepatokine	Fetuin-A	Following a single bout of exercise, obese individuals experienced an immediate rise in serum phosphofetuin-A (Ser312) levels, which returned to baseline within 24 h.	[278,290]
			This suggests that the exercise-induced decrease in fetuin-A levels may contribute to the acute health benefits of exercise observed in this context.	[279]
	Adipocyte	Adiponectin	A single 1 h bout resistance exercise enhances postprandial lipid oxidation throughout the entire body in obese men with prediabetes.	[290]
			a single session of acute exercise, regardless of intensity, leads to a notable increase in plasma adiponectin levels in sedentary, abdominally obese men.	[291]
Chronic	Myokine	Irisin	Long-term exercise decreases irisin level accompanied by reductions in body weight and body fat.	[299,300]
			cross-sectional studies have demonstrated that higher circulating irisin levels are positively associated with insulin resistance and fasting blood glucose in non-diabetic individuals.	[301,302]
	Myokine Hepatokine	FGF21	Some studies support the notion that chronic exercise, either alone or in combination with dietary intervention, can significantly reduce circulating FGF21 levels in obese or elderly individuals.	[305,306,307]
			Other studies did not observe any effect of chronic exercise on FGF21 levels in obese or diabetic patients.	[308,309]
	Adipokine	Leptin	Meta-analyses have shown that long-term aerobic, resistance, and combined exercise lead to reductions in fat mass accompanied by lower levels of leptin.	[314,315]
			Chronic exercise training for a duration of at least two weeks, whether aerobic or resistance training, resulted in decreased leptin levels in elderly postmenopausal women depending on the percentage of body fat and was observed regardless of age and sex.	[314]

HIIT: high-intensity interval training; IL: interleukin; FGF21: Fibroblast growth factor 21; T2D: type 2 diabetes.

## Data Availability

Data sharing is not applicable to this article as no new data were created or analyzed in this study.
